# The impact of the COVID-19 pandemic on all-cause mortality and life expectancy in northern Ghana: findings from the Navrongo Health and Demographic Surveillance System

**DOI:** 10.1186/s12963-025-00389-7

**Published:** 2025-06-26

**Authors:** Daniel K. Azongo, Timothy Awine, Chodziwadziwa W. Kabudula, Samuel Oladokun, Beth A. Tippett Barr, Jean Bashingwa, Bawa Ayaga, Mumuni Abu, Patrick Adum Ansah

**Affiliations:** 1 Navrongo Health Research Center, Ghana Health Service, Navrongo, Ghana; 2 MRC/Wits Rural Public Health and Health Transitions Research Unit, School of Public Health, Faculty of Health Sciences, University of the Witwatersrand, Johannesburg, South Africa; 3 Regional Institute of Population Studies, University of Ghana, Accra, Ghana; 4 Office of the Director, Nyanja Health Research Institute, Salima, Malawi

**Keywords:** Excess mortality, Mortality, COVID-19, Coronavirus, Life expectancy, Health and Demographic Surveillance Sites

## Abstract

**Background:**

Measurement of excess mortality attributable to the COVID-19 pandemic is essential for quantifying the direct and indirect effects on mortality and informing future public health control strategies. This study assessed the impact of COVID-19 on excess mortality and life expectancy at birth in rural northern Ghana.

**Methods:**

Data was obtained from the Navrongo Health and Demographic Surveillance System (NHDSS) in Ghana. We computed the number of deaths and person-years contributed from January 1, 2015, to December 31, 2021, and estimated mortality rates for each year, age group, and gender. Mortality rate differences were calculated by comparing the period before (2018–2019) and during (2020–2021) the pandemic. To estimate excess mortality, a generalised additive model was fitted to the historical data from 2015 to 2019 to predict the expected mortality rates in the COVID-19 period (2020–2021). A Cox proportional hazards model was fitted to assess the risk factors associated with mortality, adjusting for socio-demographic variables. Conventional life table techniques were used to estimate period life expectancy at birth for males and females.

**Results:**

Overall, 12,413 deaths and 1,227,115 person-years were included in the analysis. This consists of 5,930 (49%) deaths and 572,963 person-years for the pre-pandemic period (2015–2019) and 6,483 (52%) deaths and 654,153 person-years for the pandemic period (2020–2021). From 2020 to 2021, the crude mortality rate was 23.9 deaths per 1000 person-years compared with 20.7 deaths per 1000 person-years predicted if COVID-19 had not occurred. COVID-19 also caused a decline in life expectancy at birth, especially in males, by 3.4 years. In addition, the adjusted risk of dying during the pandemic was higher in males (12.1%) compared to females and higher in the 65 + years age group (69.2%) compared to the younger population.

**Conclusion:**

The COVID-19 pandemic caused an increase in deaths and a decrease in life expectancy in the NHDSS population in Ghana, particularly among men and those aged 65 years and older. These results reinforce the critical role of routine surveillance data in assessing the impact of public health emergencies such as the COVID-19 pandemic and informing public health strategies.

**Supplementary Information:**

The online version contains supplementary material available at 10.1186/s12963-025-00389-7.

## Introduction

Excess mortality estimation has become a topic of renewed interest among population scientists following the coronavirus disease 2019 (COVID-19) pandemic and its impact on population health [1, 2]. However, in many low- and middle-income countries (LMICs), epidemiological data on COVID-19 cases and deaths are either lacking or incomplete, primarily due to incomplete vital registration data [3, 4]. In this regard, several low- and middle-income countries have yet to quantify the true magnitude of excess mortality and years of life lost attributable to the COVID-19 pandemic [5]. Excess mortality analytical techniques are generally considered a more objective metric for quantifying the actual death toll of the COVID-19 pandemic by comparing observed deaths with those expected if death rates remained the same as in the pre-pandemic years [2, 6]. Several studies have shown that the COVID-19 pandemic has killed more people than reported [7]. The pandemic has also reversed recent improvements in life expectancy in many countries across the globe [8, 9]. A recent COVID-19 Excess Mortality Collaborators study estimated that the death toll in sub-Saharan Africa was 14.20 (95% uncertain interval 11.51–18.79) times higher than the officially reported deaths [10]. Populations estimated to be most affected by the pandemic were males, individuals aged 60 years and older [11], and those with underlying chronic health conditions [12, 13]. Evidence also suggests that SARS-CoV-2 infection risks and mortality rates were comparatively higher in socioeconomically disadvantaged populations [14, 15]. The disparity in the burden of COVID-19 mortality might be due to systemic inequalities, such as inadequate access to healthcare [16], housing and sanitation facilities in marginalised communities [17]. Therefore, it is important to understand the risk factors that make certain groups more vulnerable to diseases and death in order to develop more effective mitigation interventions for current and future pandemics [18].

In Ghana, the first confirmed COVID-19 cases were recorded on March 12, 2020, and the first notifiable death was reported on March 22, 2020. Since then, critical public health concerns have focused on understanding the number of deaths, which age group and gender are most affected, and the effects of mortality on the population structure and life expectancy. These statistics are crucial for informing public health control strategies and tracking the evolution of the pandemic over time [3]. Official COVID-19 morbidity and mortality data in Ghana are reported in the Ghana Health Services (GHS) health facilities. At the time of writing (August 1, 2023), the COVID-19 cases and deaths reported by the GHS stood at 171,740 and 1,462, respectively. However, these figures are likely to represent an underestimation of the actual death toll of the pandemic due to challenges in the health system, including poorly diagnosed and misclassified COVID-19 cases resulting from limited capacity and resources for testing and reporting. [19].

Additionally, there was no general screening for at-risk populations. Instead, the Ministry of Health (MoH) adopted targeted and stringent testing guidelines, including a history of travel abroad, close contact with a COVID-19 case, and any symptoms such as cough, fever, or difficulty breathing. This approach may underestimate the actual number of infected individuals [20]. Studies have also identified excess out-of-hospital mortality, suggesting that deaths outside healthcare systems were not counted in official reports, which contributes to the underestimation of the actual mortality impact of the COVID-19 pandemic [19]. In many LMICs, because the health systems databases underreport COVID-19 cases and deaths, coupled with the lack of vital registration systems, Health and Demographic Surveillance Systems (HDSS) provide an essential alternative source of epidemiological data for evaluating the impact of pandemics and quantifying the burden of diseases [21]. Studies have suggested that HDSS data are the most accurate and detailed population data routinely generated in LMICs [22].

The mortality impact of the pandemic has yet to be assessed in Ghana. To the best of our knowledge, this study is the first comprehensive published analysis of the direct and indirect effects of the COVID-19 pandemic on all-cause mortality and life expectancy in northern Ghana. Here, we describe the extent of excess mortality due to COVID-19 in the HDSS area of northern Ghana and highlight the implications for public health interventions.

## Methods

### Study design

We utilised data from a longitudinal, population-based surveillance system to investigate the impact of the COVID-19 pandemic on excess mortality and the reduction in life expectancy in rural communities of northern Ghana.

### Study setting

The study was conducted in the Navrongo HDSS area that covers the Kassena-Nankana East Municipal and Kassena-Nankana West District of the Upper East Region of Ghana, West Africa. Geographically, the surveillance area lies between latitudes 10.300 and 11.100 North and longitudes 1.10 West along the Ghana-Burkina Faso border and covers a total land area of 1,675 km^2^. The area is characterised by Guinea Savannah vegetation with two seasons: a short rainy season from June to September and a prolonged dry season from October to May. The total mid-year population for 2022 was 175,000, of whom 48 per cent are males and 35 per cent are people under 15 years (see additional files appendix 2).

The study area has a well-established primary care health system in place, which includes two hospitals, eight health centres, three private clinics, and 85 Community Health Planning Services (CHPS) clinics. Deaths in the study area are mainly caused by infectious diseases, with malaria being the leading cause of morbidity and mortality, particularly among children under five [23]. However, a recent study has shown evidence of an increase in non-communicable diseases, with neoplasms, diabetes, and cardiovascular diseases increasingly becoming important causes of death. [24]. The local economy is predominantly based on subsistence agriculture, with most of the inhabitants being peasant farmers. Educational attainment has improved over the years; however, more males are progressing to higher levels of education than females. Migration in the surveillance area is generally seasonal, with some resident populations moving to the major cities in southern Ghana in search of better employment opportunities and social services [23].

### Data source and measurements

This study uses data from the Navrongo HDSS from January 1, 2015, to December 31, 2021. The HDSS was established in 1993 to provide a platform for launching health research and monitoring the population dynamics of the two Kassena-Nankana districts in northern Ghana. Since its inception, the HDSS has routinely visited and monitored the resident population to document its health outcomes, including births, pregnancies, deaths, migrations and causes of death using verbal autopsy methods. These data have allowed for the evaluation of the mortality impact of social and health interventions and serve as a population sampling frame for various research studies conducted in the study area. Details about the HDSS platform, the type of data collected, and the data collection process have been described elsewhere [23].

Since 1993, field workers have visited all households in the area at least twice a year (every six months) to update basic demographic information. At each visit, the household census is updated, and all events of interest, including pregnancies, births and deaths are recorded. For deaths, the date of death, gender and place of death are initially recorded. These deaths are then followed by a verbal autopsy to understand the cause of death. Trained field supervisors visit households where deaths are reported. They explain the purpose of the visit to the head of the household, obtain consent, and interview the caregiver who tended to the deceased. The interviews utilised the WHO and INDEPTH-Network standardised verbal autopsy tools, which have been reviewed, validated, and widely used in multiple research settings and countries. [25]. Strict quality control measures are instituted to ensure credible data. Trained supervisors revisit three per cent of all households to cross-check the consistency and completeness of the information collected and to resolve any discrepancies and queries generated through the Household Registration System (HRS). The HRS is a structural, relational database package running on a Visual FoxPro software platform, featuring built-in data consistency checks. The software was designed as a template for generating computer programs that facilitate the collection, management, and analysis of longitudinal population studies [26].

The government of Ghana and the Ministry of Health imposed COVID-19 protocols and travel restrictions on March 16, 2020, as part of the measures to control the pandemic. However, these restrictions did not directly impact the HDSS data collection activities, as no lockdowns were enforced in the study area. Data collectors were supplied with essential personal protective equipment and trained to observe all COVID-19 protocols throughout the pandemic.

### Statistical analysis

#### Calculating the risk of mortality associated with the COVID-19 pandemic

We used longitudinal all-cause mortality data from January 1, 2015, to December 31, 2021, for the analysis. After preparing the data to enable us to estimate deaths and calculate the person-years contributed, we estimated mortality across calendar years and months. We compared crude mortality rates in 2018–2019 with those during the COVID-19 pandemic (2020–2021) by age groups, gender, rural/urban settlements and wealth index. We categorised age groups according to the WHO standardised age groupings (0–4, 5–14, 15–49, 50–64 and 65 +) to reflect the morbidity and mortality risk due to COVID-19 [27]. The socioeconomic status of households was used as a proxy for household income and wealth and was estimated based on household assets and characteristics. This was done through Principal Component Analysis (PCA) and divided into five quintiles: (1) representing the poorest, (2) very poor, (3) poor, (4) less poor, and (5) the least poor.

We subsequently defined a new variable called “COVID” to capture the COVID-19 period from March 12 2020, when the first COVID-19 case was observed in Ghana, to December 31, 2021. After initially estimating the crude risks using Cox regression models, an adjusted model was also fitted, with an interaction term between the age group and the “COVID” variable. The covariates retained in our final module include age, gender, rural/urban settlements and socioeconomic status.

#### Predicting excess mortality due to the COVID-19 pandemic

A generalised additive Poisson model was also fitted to the historical deaths data from 2015 to 2021. The data from 2015 to 2019 was used to train the model to predict the expected mortality rates for 2020 to 2021, if COVID-19 had not occurred, by gender and age group. The predicted mortality rates within the COVID-19 pandemic period (2020–2021) were then compared with the observed mortality rates to estimate the excess mortality rate attributable to the COVID-19 pandemic, along with their P-score. Thus, excess mortality rates were assessed by gender and age group.

#### Calculating the decline in period life expectancy at birth due to the COVID-19 pandemic

Conventional life table (LT) techniques- the abridged life tables model was used to estimate period life expectancy at birth and various age groups. An abridged life table is a demographic technique that summarises the probability of a person dying before entering the next hypothetical age cohort and has been used to evaluate the mortality profile of countries over time and, recently, the mortality impact of the COVID-19 pandemic on population health [28]. The estimated life expectancy was based on the deaths observed in each calendar year from 2015 to 2021. Accordingly, we calculated the life expectancy for the total population, males, females, and specific age groups (life expectancy at ages 15, 30 and 50). In addition, we calculated the age-sex-specific contribution to changes in life expectancy attributed to COVID-19 for 2020–2021. Data analysis was done using Stata version 18.0 (Stata Corporation, College Station, TX, USA) and R (statistical software).

## Results

### Descriptive results

As shown on the population pyramids in Additional File 1, the population structure of the surveillance area largely remained the same from 2015 to 2021. The pyramids show a predominantly youthful population, with a greater proportion (35%) of the population under 15 years of age.

A total of 12,413 deaths and 1,227,116 person-years were included in the analysis, and out of this, 5,930 (48%) and 572,963 person-years occurred during the reference period 2015–2019, while 6483 deaths (52%) and 654,153 person-years occurred during the pandemic period of 2020–2021. The number of deaths and the person-years contribution by age group, gender, rural/urban settlement and socio-economic status between 2015 and 2021 are presented in Table 1. From 2015 to 2020, the number of deaths steadily declined. However, during the peak of the COVID-19 pandemic, the mortality increased dramatically. In 2019, the number of deaths and person-years was 1574 (165.7 deaths per 1000 person-years), but in 2021, the absolute death toll rose to 2656 (241.1 deaths per 1000 person-years). Among the 65 + age group, the number of deaths and person-years increased from 728 and 11.8 deaths per 1000 person-years in 2019 to 1,329 and 17.6 deaths per 1000 person-years in 2021, respectively.

**Table 1:** Distribution of deaths and person-years at risk of death by demographic variables for the baseline versus COVID-19 period in the Navrongo HDSS area

Variables/pry (deaths)	Number of deaths (rate per 1000/person-years)						
	2015	2016	2017	2018	2019	2020	2021
Distribution of deaths and person-years at risk by demographic variables from 2015–2021 in the Navrongo HDSS site							
All ages	1647(160.0)	1548(163.7)	1706(165.2)	1582(165.3)	1574(165.7)	1700(166.1)	2656(241.1)
Age group							
0–4	203(19.3)	126(19.7)	126(20.0)	106(20.4)	109(20.3)	84(20.0)	128(29.0)
5–14	35(38.0)	44(38.6)	48(39.0)	39(39.2)	42(39.2)	51(39.2)	51(56.9)
15–49	227(27.0)	341(76.1)	407(76.7)	351(76.2)	336(76.1)	359(76.2)	538(110.6)
50–64	324(17.5)	351(17.7)	354(17.9)	366(18.0)	359(18.3)	371(18.7)	610(27.1)
65+	792(11.3)	686(11.6)	771(11.6)	720(11.6)	728(11.8)	835(12.0)	1329(17.6)
Gender							
Males	916(76.4)	881(78.2)	1007(79.1)	891(79.1)	889(79.2)	975(79.5)	1556(115.1)
Females	731(83.5)	667(85.5)	699(86.1)	691(86.3)	685(86.5)	725(86.7)	1100(126.0)
Location							
Rural	1430(132.8)	1331(136.0)	1474(137)	1337(136.7)	1326(137.1)	1337(136.7)	2271(199.9)
Urban	191(24.3)	188(24.5)	203(24.4)	209(24.4)	207(24.3)	209(24.4)	326(35.4)
Wealth quintiles							
Poorest	512(42.6)	464(43.5)	520(43.6)	483(43.2)	445(43.1)	490(43.1)	766(61.9)
Very poor	423(35.2)	380(36.0)	439(36.2)	372(36.1)	366(36.1)	410(36.1)	676(52.0)
Poor	311(32.0)	325(32.7)	333(33.1)	342(33.0)	324(33.1)	360(33.2)	534(48.4)
Less poor	229(26.1)	228(26.6)	243(26.9)	227(27.0)	260(27.2)	243(27.5)	395(40.5)
Least poor	144(21.1)	122(21.4)	141(21.5)	121(21.6)	136(21.7)	158(21.9)	223(32.2)

**Fig. 1:**
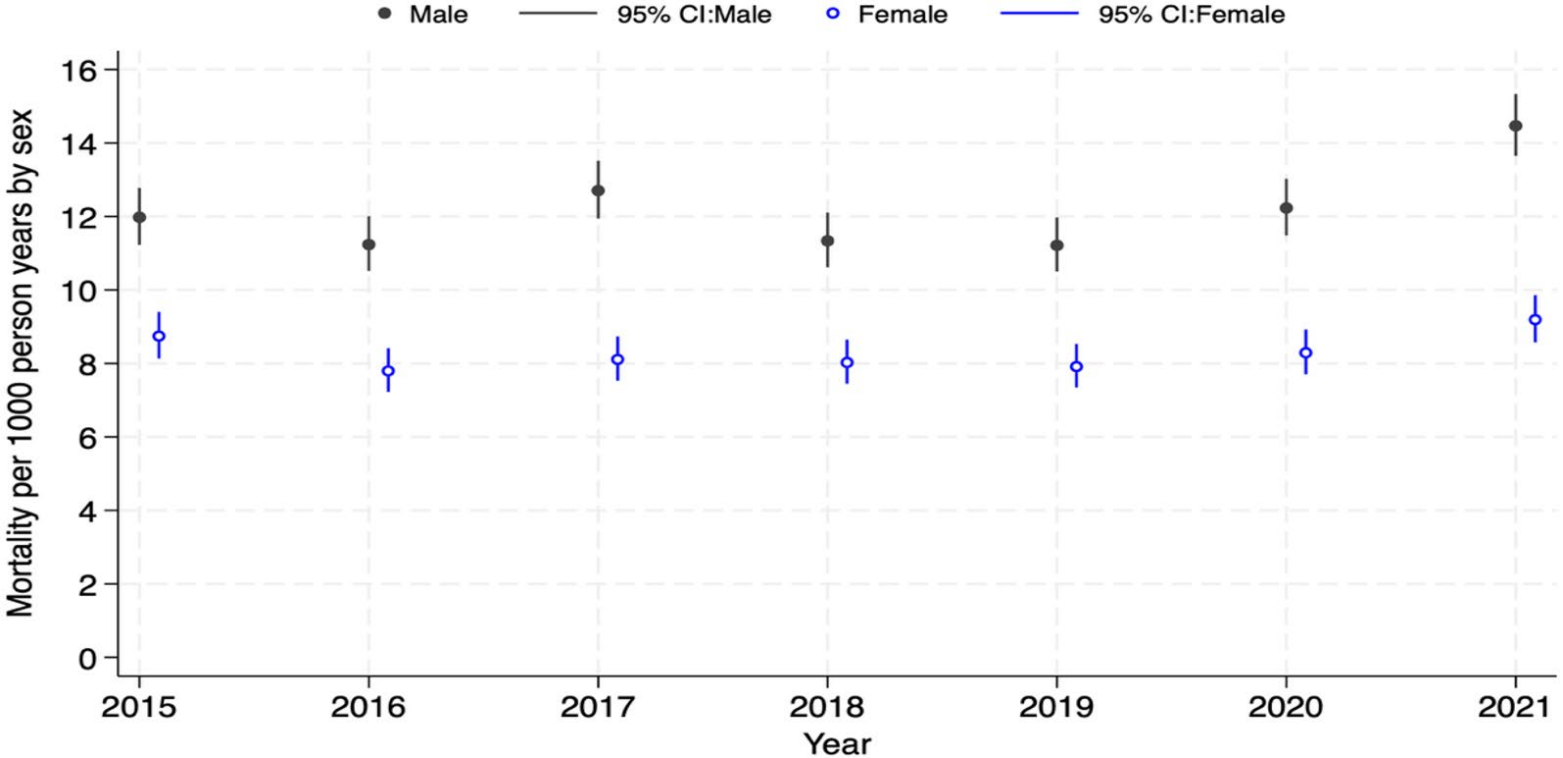
Trends in all-cause mortality by males and females from 2015 to 2021 in the Navrongo HDSS study area

**Fig. 2:**
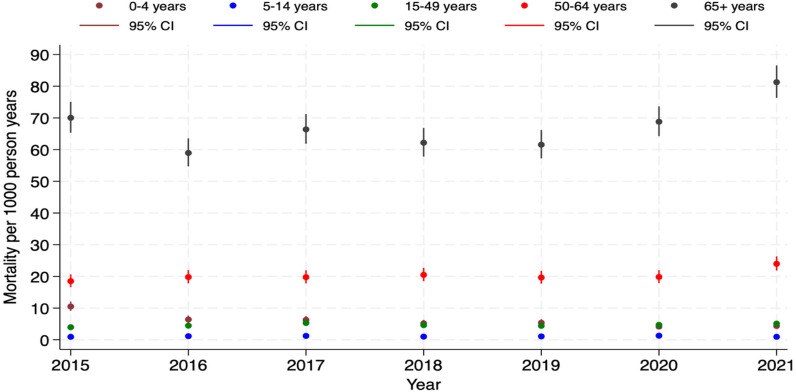
Trends in all-cause mortality rates by age group (0–4, 5–14, 15–49, 50–64 and 65 + years) from 2015 to 2021 in the Navrongo HDSS study area

A trend analysis of the mortality pattern shows that males have had higher mortality rates since 2015 (Fig. 1). Additionally, there was a significant increase in mortality rates from 2020, particularly for those aged 65 and over, even though trends in mortality rates remained consistent from 2015 to 2019, as illustrated in Fig. 2.

### Analysis of the contribution of COVID-19 to excess mortality

Following this analysis, we further assessed the mortality incidence rate by age group, gender and socio-economic status between the period immediately before the COVID-19 outbreak (2018–2019) and during the pandemic (2020–2021), illustrated in Table 2. Apart from the 0–4 years and 5–14 years age groups, all other age groups experienced an increase in the incidence rate of mortality in the pandemic period 2020–2021. The mortality incidence rate difference increased progressively with age from 15 years, with the most noticeable incidence rate difference observed among the 65 + years age group 11.19, (95% CI: 6.77–16.62) per 1000 person-years, followed by the 50–64 years age group 1.47 (95% CI: − 0.51 to 3.35) per 1000 person-years. By gender, males experienced an incidence rate difference of 1.76 (95% CI: 1.03–2.49) per 1000 person-years, while the poorest wealth quintiles experienced an incidence rate difference of 1.22 (95% CI: 0.26–2.18) per 1000 person-years.

**Table 2:** Comparison of mortality incidence rates between the period (2018–2019) and the COVID-19 period (2020–2021) and the crude incidence rate difference

Variable	Incidence 2018–2019			Incidence rate 2020–2021			Incidence rate difference			
	IR per 1000	Lower bound 95% CI	Upper bound 95% CI	IR per 1000	Lower bound 95% CI	Upper bound 95% CI	IRD per 1000	Lower bound 95% CI	Upper bound 95% CI	P value *
Comparison of mortality incidence rates between the reference period (2018–2019) and the COVID-19 period (2020–2021) and the incidence rate difference										
Age group										
0–4	5.3	4.6	6	4.3	3.8	5	−0.95	−1.86	−0.03	< 0.001
5–14	1	0.8	1.3	1.1	0.9	1.3	0.03	−0.28	0.33	
15–49	4.5	4.2	4.9	4.8	4.5	5.1	0.29	−0.17	0.75	
50–64	20	18.6	21.5	21.5	20.2	22.8	1.47	−0.51	3.45	
65+	61.8	58.7	65	73	69.9	76.1	11.19	6.77	15.62	
Gender										
Males	11.2	10.7	11.8	13	12.5	13.5	1.76	1.03	2.49	0.016
Females	8	7.6	8.4	8.6	8.2	9	0.62	0.04	1.19	
Location										
Rural	9.7	9.4	10.1	11	10.7	11.4	1.32	0.81	1.84	0.116
Urban	8.5	7.8	9.4	8.9	8.1	9.7	0.34	−0.78	1.45	
Wealth quintiles										
Poorest	10.7	10.1	11.5	12	11.3	12.6	1.22	0.26	2.18	0.261
Very poor	10.2	9.5	11	12.3	11.6	13.1	2.1	1.06	3.14	
Poor	10.1	9.3	10.9	10.9	10.3	11.7	0.87	−0.17	1.92	
Less poor	9	8.2	9.8	9.4	8.7	10.1	0.41	−0.67	1.49	
Least poor	5.9	5.2	6.7	7	6.4	7.8	1.11	0.1	2.13	

*Test of homogeneity

**Fig. 3:**
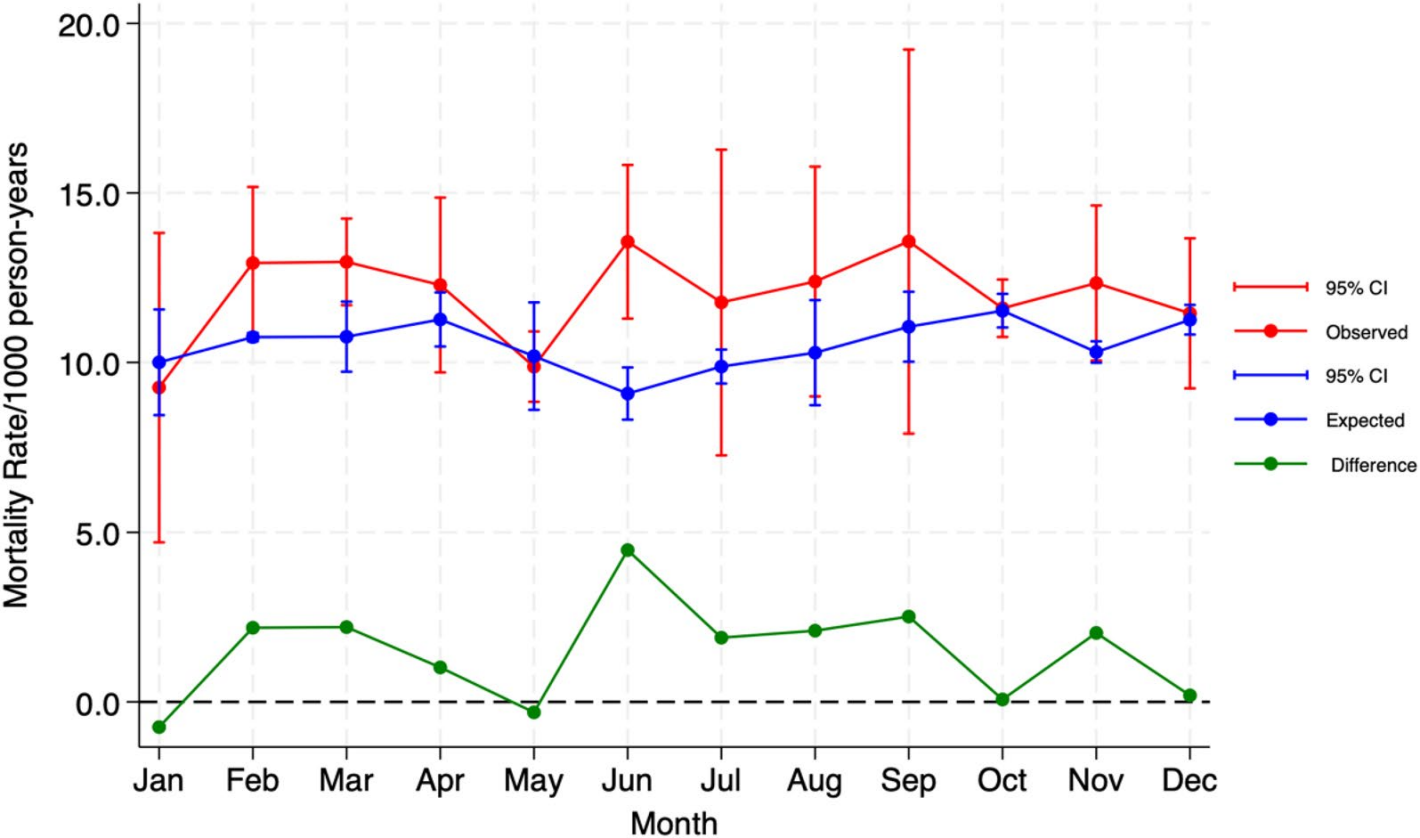
Monthly trends in excess mortality during the COVID-19 pandemic (2020–2021) (using outputs from the predictions of the Poisson generalised additive model) in the Navrongo HDSS study area

Similarly, the monthly mortality incidence rate (MIR) difference (deviation in mortality rate from the expected level compared to the observed rate) during the COVID-19 period (2020–2021) is illustrated in Fig. 3. During certain months, the mortality incidence rates of COVID-19 were higher than expected. Specifically, the incidence rate difference was lower in January and May, indicating a slightly lower number of observed deaths. However, the incidence rates increased in the remaining months, peaking in June at 14 deaths per 1000 person-years, which is higher than expected.

**Fig. 4:**
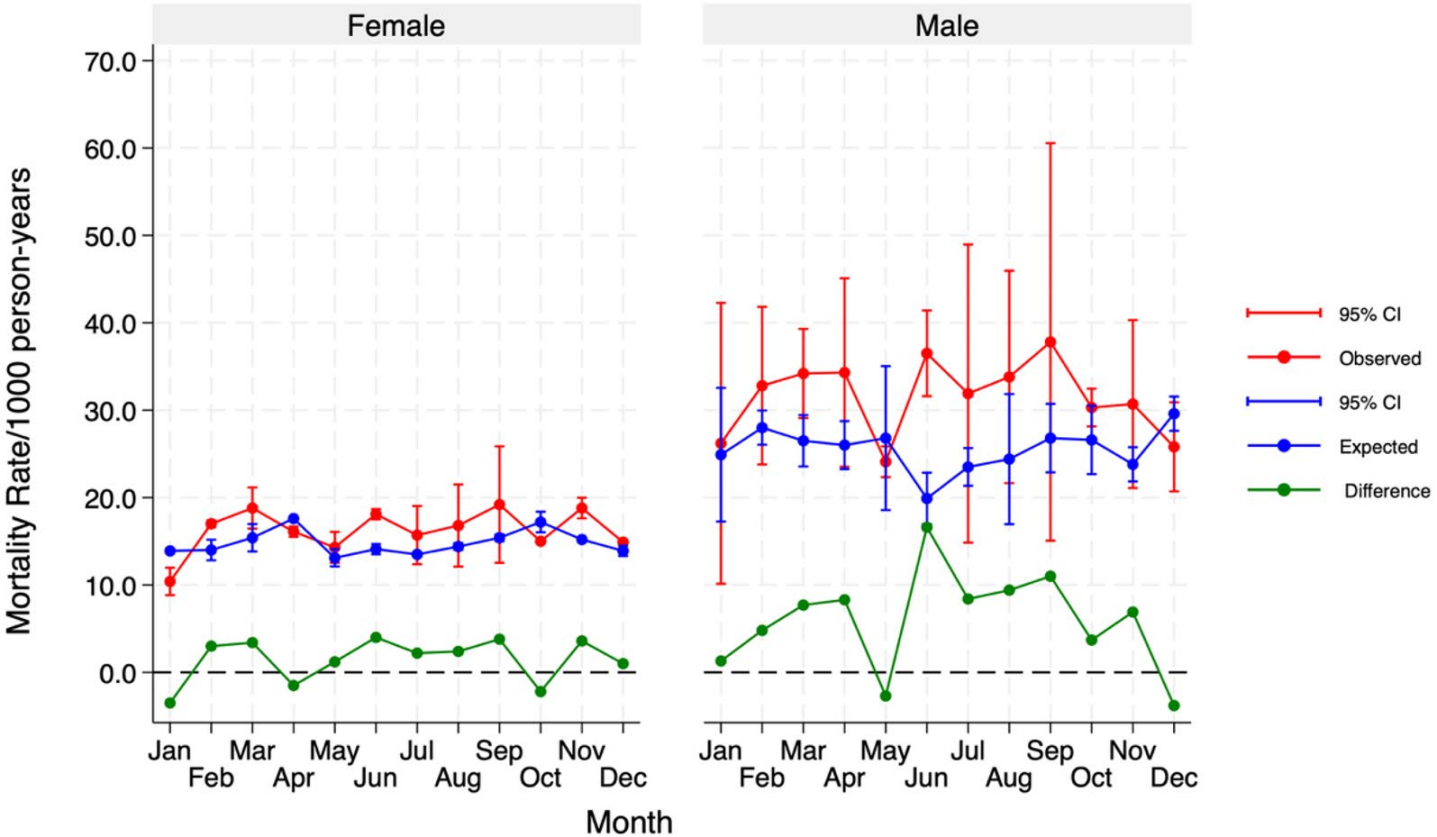
Monthly trends in excess mortality by gender during the COVID-19 pandemic (2020–2021) (using outputs from the predictions of the Poisson generalised additive model) in the Navrongo HDSS study area

A further analysis of the monthly trends of excess mortality by age groups was consistent with the previous results (Fig. 4). The observed mortality rates among the 50–64 age group were much higher than expected in the COVID-19 period (Fig. 3). While females had relatively normal mortality rates, males showed a much higher deviation from expected mortality levels (Fig. 4). On the other hand, children aged 0–4 years old experienced the lowest number of observed deaths during the pandemic than what would have been expected under normal circumstances, as illustrated in supplementary information (see Fig. 2).

Between January 2020 and December 2021, the all-cause mortality rate was 12.0 per 1000 person-years for all age groups and genders. This means that for every 1000 people, 12 individuals died during this period. In contrast, the expected all-cause mortality rate for the same period was 10.3 per 1000 person-years, resulting in an excess mortality rate of 1.7 deaths per 1000 person-years. The highest excess mortality rates were observed in males (5.9 per 1000 person-years) and adults aged 65 years and older (16.6 per 1000 person-years). The only age group that recorded reduced excess mortality rates were the 0–4 age group, with a decrease of − 2.3 deaths per 1000 person-years. This means that the number of deaths in this age group was lower than expected. The p-scores (i.e., the percentage by which observed deaths exceed expected deaths) also show that males (18.7%) and older adults aged 65 + (19.9%) experienced the highest proportions of excess mortality, as illustrated in Table 3.

**Table 3:** Distribution of excess mortality rates by age groups and gender attributed to COVID-19 (using outputs from the predictions of the Poisson generalised additive model) in the Navrongo HDSS area (2020–2021)

Variables	Observed rate per 1000/prys	Expected rate per 1000/prys	Excess mortality rate 1000/prys	P-Score (%)
All ages	12	10.3	1.7	12.5
Age				
0–4	4.5	6.8	−2.3	−51.1
May-14	1.1	1	0.1	9.1
15–49	5.1	4.7	0.4	7.8
50–64	25.2	21.5	3.7	14.7
65+	83.5	66.9	16.6	19.9
Gender				
Females	16.3	14.8	1.5	9.2
Males	31.5	25.6	5.9	18.7

### The effects of COVID-19 on period life expectancy at birth

**Fig. 5:**
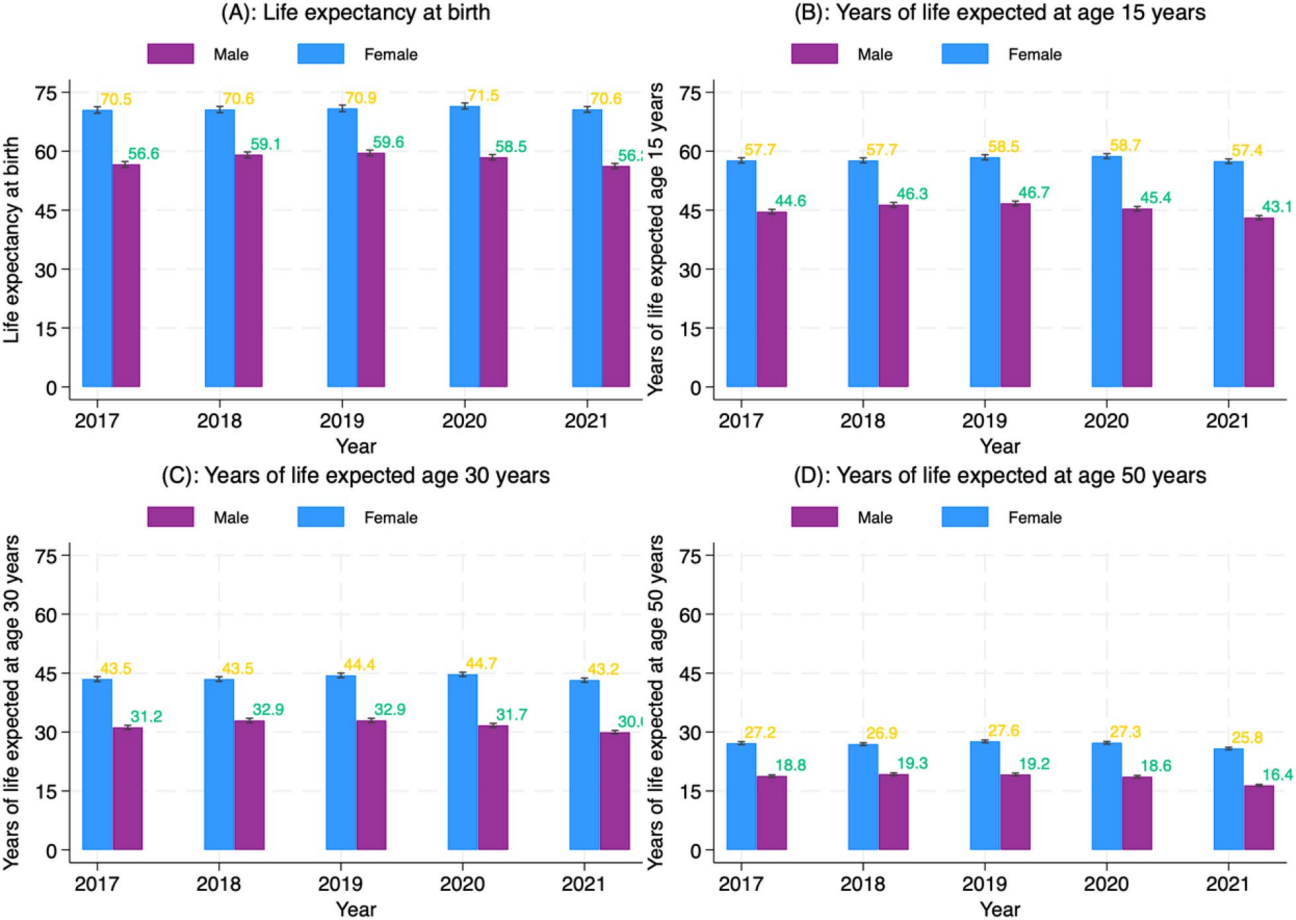
Life expectancy at birth and age 15, 30, and 50 years and by gender from 2015–2021 in the Navrongo HDSS study area

A second analysis was performed to assess the impact of the COVID-19 pandemic on life expectancy (LE) during the period 2020–2021. Our findings indicate that the pandemic has slightly reduced life expectancy in the study area. Figure 5 illustrates the trends and changes in life expectancy for various age groups and by sex, both before and during the pandemic. In 2015, the average life expectancy at birth for females was 68.5 years, and for males, it was 58.6 years. Between 2015 and 2019, there was a slight increase in life expectancy for both males and females. In 2019, the life expectancy for females was 70.9 (95% CI: 70.1–71.7) years, while for males, it was 59.6 (95% CI: 58.9–60.3). However, in 2021, the figures decreased to 70.6 (95% CI: 69.9–71.4) years for females and 56.2 (95% CI: 55.6–56.9) years for males (refer to Fig. 5a). The decrease in life expectancy as a result of the effects of the pandemic was more significant for males, with a decline of 3.4 years, compared to females, who experienced a decline of only 0.3 years.

The decrease in life expectancy was more pronounced among individuals aged 50 years and above, particularly among males in this age range (Fig. 5d). For example, the LE for males in 2019 at age 50 and above was 19.2 years, but this declined to 16.4 years in 2021, resulting in a loss of approximately 2.8 years in LE as of the end of the pandemic. The decline in life expectancy (LE) is particularly noticeable among those aged 25 to 65 years, who experienced a reduction in LE of 3.4 years and 5.7 years, respectively. Moreover, men have had a more significant decrease in life expectancy than women, with a difference of 2.16 years and 1.50 years, respectively.

**Fig. 6:**
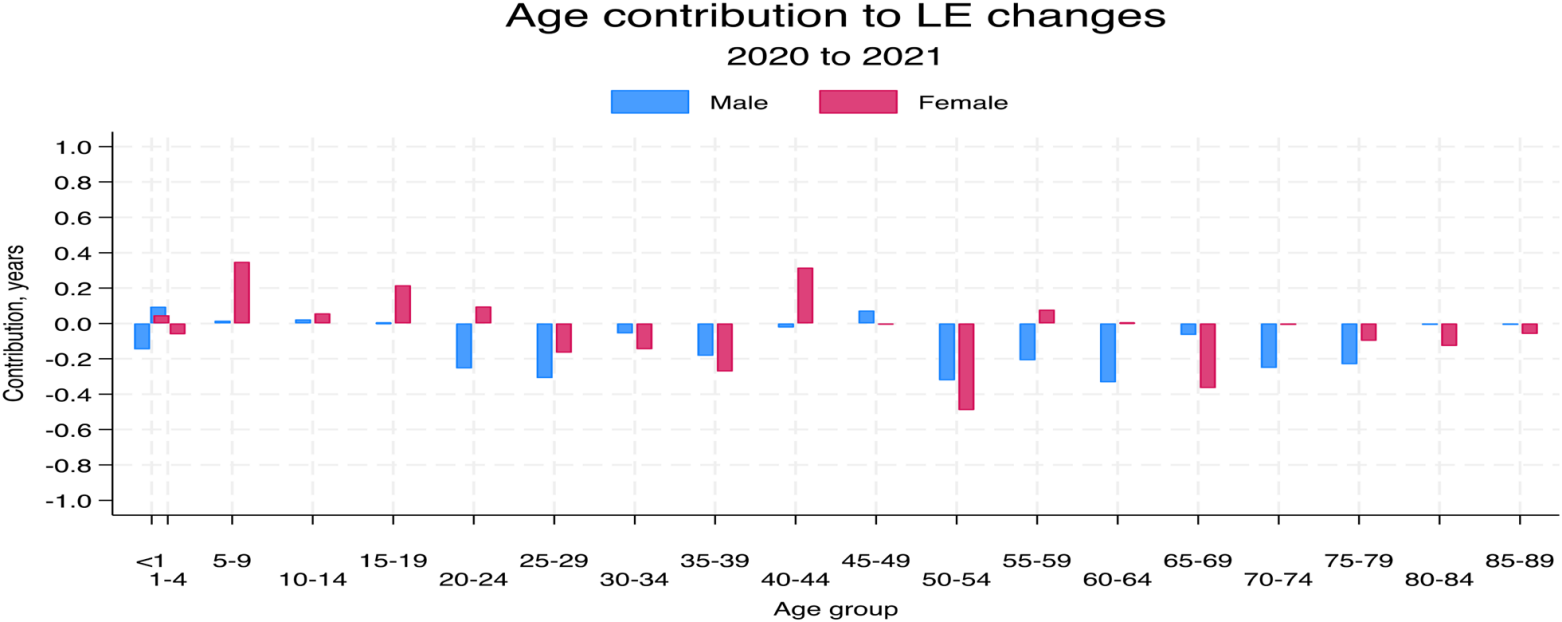
Age contributions to changes in life expectancy from 2019 to 2021 in the Navrongo HDSS study area

We further examined how different age groups contributed to changes in life expectancy during the pandemic (refer to Fig. 6). The results indicate that the older age groups and male gender contributed negatively to overall changes in LE compared to the younger age groups and females. A striking finding was the contribution of male adults aged 20–29 years to the reduction in life expectancy.

### The risk of death and associated socio-demographic factors

The third set of analyses examined the risk of death associated with socio-demographic factors. The results of the Cox proportional hazards model for the crude hazards and the adjusted risks of mortality are shown in Table 4. The crude risk of death in the pandemic period was estimated to be 13.0% [95% CI: 09.0–18.0%] higher than the two years before the pandemic (2018–2019).

**Table 4:** Socio-demographic factors associated with all mortality during the COVID-19 pandemic in the Navrongo HDSS study area (2020–2021)

Variables	Unadjusted Cox regress model				Adjusted Cox regression model			
	Haz. ratio	Lower bound 95% CI	Upper bound 95% CI	P value	Haz. ratio	Lower bound 95% CI	Upper bound 95% CI	P value
Age group								
0–4	ref							
5–14	0.18	0.16	0.21	**< 0.001**	0.16	0.14	0.19	**< 0.001**
15–49	0.78	0.72	0.84	**< 0.001**	0.68	0.62	0.75	**< 0.001**
50–64	3.4	3.17	3.69	**< 0.001**	3.11	2.84	3.4	**< 0.001**
65+	11.3	10.51	12.11	**< 0.001**	10.24	9.43	11.13	**< 0.001**
Gender								
Females	ref							
Males	1.47	1.42	1.52	**< 0.001**	1.98	1.91	2.05	**< 0.001**
Place of residence								
Rural	ref							
Urban	0.8	0.76	0.85	**< 0.001**	1.14	1.06	1.21	**< 0.001**
Wealth quintiles								
Poorest	ref							
Very poor	0.99	0.95	1.05	0.964	1.02	0.97	1.07	0.383
Poor	0.9	0.85	0.94	**< 0.001**	0.98	0.93	1.03	0.422
Less poor	0.79	0.75	0.83	**< 0.001**	0.91	0.86	0.97	**0.002**
Least poor	0.56	0.53	0.6	**< 0.001**	0.72	0.67	0.79	**< 0.001**
Age Group # COVID*								
0–4	ref							
5–14					1.44	1.08	1.93	**< 0.001**
15–49					1.59	1.32	1.9	**< 0.001**
50–64					1.7	1.42	2.04	**< 0.001**
65+					1.76	1.49	2.08	**< 0.001**
COVID**								
2018–2019	ref							
2020–2021	1.13	1.09	1.18	**< 0.0001**	0.69	0.59	0.81	**< 0.001**

*Age group#COVID-19 period (Interaction term for COVID-19 and age group)

**The COVID-19 period starts from March 2020 to Dec 202

Bold values are used to highlight hazard ratio & 95% confidence Interval

Hazard ratios decreased with an increasing wealth index. While the relative hazard among the very poor versus the poorest was 0.99 [95% CI: 0.95–1.05], the least poor were 44.0% [95% CI: 40.0–47.0%] more protected compared to the poorest, and the results were statistically significant.

We observed that the likelihood of dying was 20% lower for those living in urban areas compared to those in rural areas, 0.8 [95% CI: 0.75–0.85]. However, men had a higher risk of death than women, with a relative hazard of 47.0% (95% CI: 42.0–52%). In terms of age, individuals aged 65 and over had the highest risk of death, with a risk of 11.3 (95% CI: 10.5–12.1), compared to those in the 0–4 age group, as illustrated in Table 4.

After adjusting for age group, gender, residence location, and wealth index and taking into account the impact of the COVID-19 period on different age groups (fitting an interaction between the COVID-19 period and age groups), the hazard ratio during the COVID-19 period was 0.7 [95% CI: 0.6–0.8] compared to the two years before the first case was reported. The relative risks among different wealth indices remained consistently significant as the crude risks; however, the relative hazard among males increased significantly, doubling to 2.0 [95% CI: 1.9–2.1] in the adjusted model. The hazard ratios for different age groups were all statistically significant: 18.0 [95% CI: 14.1–23.2] for the 65 + age group, 5.3 [95% CI: 4.0–6.9] for 50–64 years, 1.1 [95% CI: 0.8–1.4] for 15–49, and 0.2 [95% CI: 0.2–0.4] for the 4–14 age group.

## Discussion

The population-level mortality impact of the COVID-19 pandemic has not been systematically assessed in many low- and middle-income countries, including Ghana. This study used longitudinal population-based data on all-cause mortality from 2015 to 2021 to assess the effects of COVID-19 on all-cause mortality and life expectancy at various ages in the two Kassena-Nankana Districts of northern Ghana. From January 2020 to December 2021, we found that the cumulative excess mortality rate was 1.7 deaths per 1000 person-years for both sexes combined and 5.9 and 1.5 deaths per 1000 person-years among males and females, respectively. During the same period, life expectancy decreased by 0.3 years for females and 3.4 years for males compared to historical trends. The study found that males and persons aged 65 years and above had a higher mortality risk than other age groups. This is consistent with historical trends, as males tend to have higher mortality rates and lower life expectancy and are not just exceptional to the COVID-19 pandemic [29]. The higher mortality risk in males can be attributed to biological differences, lifestyle choices, and occupational hazards [30]. However, there are several possible explanations regarding why the elderly have a higher excess mortality rate than other age groups. One argument is that mortality rates increase with age. As individuals age, they are more likely to develop chronic health conditions and experience a decline in physiological function, which can contribute to a higher risk of mortality [31, 32]. Additionally, studies have shown that the COVID-19 pandemic disproportionately affected those with underlying chronic health conditions, which are more prevalent among the elderly [12]. An alternative explanation for these findings is that older adults are more likely to have poor health conditions, including comorbidities such as cardiovascular disease, diabetes, and respiratory infections, which can increase their vulnerability to morbidity and mortality due to SARS-CoV-2/COVID-19 [13].

Conversely, the COVID-19 pandemic seems to have a lesser impact on child mortality, resulting in a deficit in excess mortality. Although it is difficult to explain this result, a recent study suggests that infants and young children’s immune systems are better equipped to fight off SARS-CoV-2 infection as young children develop robust and sustained cross-reactive immune responses to SARS-CoV-2 infection that lasted far longer than adults [33]. The findings could also reflect Ghana's sustained public health efforts to reduce preventable causes of child mortality. These efforts include implementing the Expanded Programme on Immunization to improve childhood vaccination coverage, including the Bacille Calmette-Guérin (BCG) vaccine. BCG vaccinations have been suggested to provide non-specific protection against viral infection, including SARS-CoV-2 [34, 35]

Our results also show a positive association between COVID-19 mortality and socioeconomic status. These findings are broadly in line with the recent literature on the uneven health risks of the COVID-19 pandemic and highlight how economically deprived populations are more vulnerable to the burden of diseases [36]. Studies from various countries have reported that living and working conditions, including crowded housing conditions, travel to higher-risk areas, and poor healthcare systems, can increase the risk of infection [37, 38]. A study by Wachtler and colleagues (2020) on socio-economic inequalities in the risk of SARS-CoV-2 infection in Germany found higher infection risk in regions with low socioeconomic status [39]. These findings show that socioeconomically disadvantaged individuals may be more susceptible to the COVID-19 pandemic, which can increase pre-existing health inequalities in those regions.

Furthermore, the analysis revealed fluctuating patterns of excess mortality rates across different phases of the pandemic and among various age groups, with some months experiencing levels below the expected mortality rates and others showing spikes far above the expected levels. Although these patterns have been reported in other studies, the specific factors behind the fluctuations remain unclear [40]. However, they can be attributed to a combination of factors, including the direct impact of COVID-19 infections, indirect effects of the pandemic on healthcare systems, including access to care, and other underlying causes of death [41]. A recent study reported that Ghana recorded four major COVID-19 transmission waves, with the highest positivity rate reported in the fourth wave [42], which was exacerbated by the harsh harmattan weather conditions [43].

Our findings have supported the position that the impact of the COVID-19 pandemic has been grossly underestimated in many low- and middle-income countries [2, 10]. These results match those observed in Ghana by Asumanu E. et al. (2021), who conducted a post-mortem study to investigate the causes of death and SARS-CoV-2 in deceased bodies brought to a health facility and found that SARS-CoV-2 was present in 12.4% of the cases and was responsible for inducing death in 2.5% (4/161) of the cases [44].

Our analysis has strengths, including high-quality all-cause mortality data and the application of standard approaches for estimating excess mortality. However, while these findings are likely to mirror the effect of the pandemic nationwide, they must be interpreted with caution because they only reflect patterns of mortality in a rural region in Northern Ghana with a population of about 175,000 people.

## Conclusion

Building on existing literature, the present study is the first to comprehensively explore the effects of the COVID-19 pandemic on mortality and life expectancy in a rural setting in Northern Ghana. We demonstrated that the COVID-19 pandemic has reversed favourable historical mortality and life expectancy trends in the adult population. These findings align with the broader understanding that the COVID-19 pandemic has, directly and indirectly, affected mortality rates worldwide and reduced gains in life expectancy. The most prominent finding emerging from this study is that even in rural areas with dispersed population settlements but with limited access to healthcare, COVID-19 has increased overall mortality levels and led to losses in life expectancy, particularly among the elderly population, males and socioeconomically disadvantaged groups. Overall, the findings suggest that the pandemic can reverse progress made in public health in Ghana. It is, therefore, crucial for the government and health authorities to provide adequate resources and equipment for the health system, conduct health education campaigns on mask-wearing and handwashing, and prioritise vaccination efforts for vulnerable groups, such as the elderly and working-age population. Investment in disease and mortality surveillance is also necessary for gathering data to inform health policies and control strategies, particularly during future pandemics, as has been outlined in the WHO strategic framework for emergency preparedness [45].

The present study also lays the foundation for future research to understand the impact of COVID-19 on cause-specific mortality and years of life lost in Ghana to gain insights into the burden of disease and geographic areas that have been disproportionately affected by the pandemic. This can be achieved by analysing cause-of-death data and using spatial analytical techniques. From a public health standpoint, understanding risk factors for disease burden is vital for policy decisions during pandemics. This knowledge can inform current and future public health control strategies in Ghana and similar regions elsewhere.

## Supplementary Information

Additional file 1: Population pyramid of the Navrongo HDSS area from 2015 to 2021 in the Navrongo HDSS study area

Additional file 2: Graphs showing monthly trends in excess mortality by age group during the COVID-19 pandemicin the Navrongo HDSS study area

## Data Availability

Data used in this paper is made available on the MRC/Wits Agincourt Research Unit Data Repository (https://data.agincourt.co.za/index.php/catalog/339). Data containing other covariates used in the analysis reported in this manuscript can be accessed through a formal request to the corresponding author.
