# Supplementary material for: The impact of the COVID-19 pandemic on all-cause mortality and life expectancy in northern Ghana: findings from the Navrongo Health and Demographic Surveillance System
Source: Popul Health Metr. 2025 Jun 26;23(Suppl 2):31. doi: 10.1186/s12963-025-00389-7 (PMC12199484; doi:10.1186/s12963-025-00389-7)
Supplement: Supplementary file 1 — Additional file 1: Population pyramid of the Navrongo HDSS area from 2015 to 2021 in the Navrongo HDSS study area [file 12963_2025_389_MOESM1_ESM.docx]

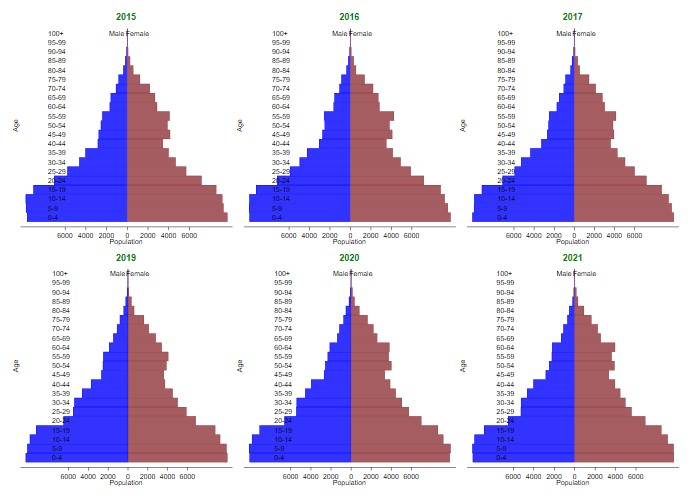


### Additional file 1: Population pyramid of the Navrongo HDSS area from 2015 to 2021 in the Navrongo HDSS study area.
